# Myricetin alleviates diabetic cardiomyopathy by regulating gut microbiota and their metabolites

**DOI:** 10.1038/s41387-024-00268-4

**Published:** 2024-03-12

**Authors:** Jinxiu Zhu, Zhijun Bao, Zuoqi Hu, Shenglin Wu, Cuihong Tian, Yueran Zhou, Zipeng Ding, Xuerui Tan

**Affiliations:** 1 Institute of Clinical Electrocardiology, the First Affiliated Hospital of Shantou University Medical College, 515041 Shantou, Guangdong China; 2 Longgang Maternity and Child Institute of Shantou University Medical College (Longgang District Maternity & Child Healthcare Hospital of Shenzhen City), 518172 Shenzhen, Guangdong China; 3 Department of Cardiology, the First Affiliated Hospital of Shantou University Medical College, 515041 Shantou, Guangdong China; 4 Clinical Research Center, the First Affiliated Hospital of Shantou University Medical College, 515041 Shantou, Guangdong China

**Keywords:** Diabetes complications, Cardiovascular diseases

## Abstract

**Background:**

The gut microbiota is involved in the pathogenesis of diabetic cardiomyopathy (DCM). Myricetin protects cardiac function in DCM. However, the low bioavailability of myricetin fails to explain its pharmacological mechanisms thoroughly. Research has shown that myricetin has a positive effect on the gut microbiota. We hypothesize that myricetin improves the development of DCM via regulating gut microbiota.

**Methods:**

DCM mice were induced with streptozotocin and fed a high-fat diet, and then treated with myricetin by gavage and high-fat diet for 16 weeks. Indexes related to gut microbiota composition, cardiac structure, cardiac function, intestinal barrier function, and inflammation were detected. Moreover, the gut contents were transplanted to DCM mice, and the effect of fecal microbiota transplantation (FMT) on DCM mice was assessed.

**Results:**

Myricetin could improve cardiac function in DCM mice by decreasing cardiomyocyte hypertrophy and interstitial fibrosis. The composition of gut microbiota, especially for short-chain fatty acid-producing bacteria involving Roseburia, Faecalibaculum, and Bifidobacterium, was more abundant by myricetin treatment in DCM mice. Myricetin increased occludin expression and the number of goblet cells in DCM mice. Compared with DCM mice unfed with gut content, the cardiac function, number of goblet cells, and expression of occludin in DCM mice fed by gut contents were elevated, while cardiomyocyte hypertrophy and TLR4/MyD88 pathway-related proteins were decreased.

**Conclusions:**

Myricetin can prevent DCM development by increasing the abundance of beneficial gut microbiota and restoring the gut barrier function.

## Introduction

It is expected that the number of people with diabetes will rise to 400 million in 2030, and ~80% of them will experience cardiovascular disease [1–3]. Diabetic cardiomyopathy (DCM), one of the major complications of diabetes, poses a high risk of mortality to patients with diabetes [4]. Multiple complex mechanisms involving unbalanced energy metabolism, oxidative stress, inflammation, and mitochondrial dysfunction contribute to the development of DCM [5, 6]. Particularly, the activation of inflammatory cells and signals, and the release of inflammatory cytokines, accelerate the process of cardiomyocyte hypertrophy and cardiac fibrosis in the diabetic heart [7–9]. Therefore, anti-inflammation has been considered an important strategy for treating DCM [10].

The gut microbiota maintains a symbiotic relationship with the host and plays a critical role in numerous physiological functions involving nutrient metabolism, immunomodulation, and maintenance of the gut barrier [11, 12]. Disruption of gut microbiota has been reported to be associated with cardiovascular disease and autoimmune disease, e.g., inflammatory bowel disease, diabetes, rheumatoid arthritis, and multiple sclerosis [13, 14]. Gut microbiota composition is altered in type 2 diabetes. The abundance of butyrate-producing bacteria, for example, *Roseburia intestinalis* and *Faecalibacterium prausnitzii*, are decreased in patients with type 2 diabetes, while conditional pathogens, such as *Escherichia coli*, some *Clostridium* species, *Bacteroides caccae* and *Eggerthella lenta* are enriched [15]. Due to the increased intestinal permeability in type 2 diabetes, gut microbial products can translocate into the blood, resulting in metabolic endotoxemia. For example, lipopolysaccharide (LPS), a key mediator of endotoxemia, can activate an inflammatory response through the toll-like receptor 4/myeloid differentiation factor 88 (TLR4/MyD88) signaling pathway [16]. By interacting with TLR4, LPS can damage cardiomyocytes [17] and block TLR4 signaling, resulting in cardiac dysfunction [18, 19].

Myricetin, a polyphenolic compound, has diverse pharmacological activities, including antioxidative, anti-inflammatory, anti-atherosclerotic, and immunomodulatory properties [20]. It can alleviate oxidative stress and inflammation by suppressing the nuclear factor-κB (NF-κB) pathway in DCM [21]. However, due to its extremely low bioavailability, it is hard to adequately explain the therapeutic efficacy of myricetin [22], suggesting the existence of other potential mechanisms of myricetin action. Polyphenols are the underlying substrates of gut microbiota, and regulating gut microbiota is one of the mechanisms of their physiological effects [22]. Myricetin supplementation has been shown to regulate the gut microbiota in rats with non-alcoholic fatty liver disease (NAFLD) by increasing the abundance of butyric acid-producing bacteria and decreasing plasma LPS that activates TLR4, leading to decreased inflammation [23]. Dihydromyricetin, a myricetin derivative, can ameliorate inflammatory bowel disease by regulating intestinal bacteria-associated bile acid metabolism [24]. Therefore, myricetin can regulate gut microbiota and reduce inflammation. However, whether myricetin can ameliorate DCM by regulating gut microbiota remains unclear.

Therefore, we hypothesized that myricetin could inhibit the development of DCM by mediating gut microbiota. A streptozotocin (STZ)-induced DCM mouse model followed by a fecal microbiota transplantation (FMT) experiment was constructed and demonstrated that myricetin could alleviate DCM via regulating the composition of gut microbiota and restoring intestinal barrier function and the re-establishment of beneficial gut microbiota is essential to maintain the high bioactivity of myricetin.

## Materials and methods

### Chemicals

Myricetin was purchased from Chengdu Herbpurify Co. Ltd. (Chengdu, China), and its purity was >98%, as determined by high-performance liquid chromatography (HPLC). Vancomycin, neomycin sulfate, metronidazole, and ampicillin were obtained from the Beijing Vital River Laboratory Animal Technology Co., Ltd. (Beijing, China).

### Animals and treatments

Four-week-old male C57BL/6J mice were purchased from the Beijing Vital River Laboratory Animal Technology Co., Ltd. (Beijing, China) and housed at 22–26 °C in a specific pathogen-free standard laboratory environment on a 12-h light/dark cycle with free access to a normal diet and water.

After acclimating for a week, the mice were randomly assigned to four groups (*n* = 10 per group): control (CON) (fed a normal diet for 4 weeks, then treated with the STZ vehicle for 5 days followed by intragastric administration of myricetin vehicle twice a day for 16 weeks), myricetin (M) (fed a normal diet for 4 weeks and treated with the STZ vehicle for 5 days followed by intragastric administration of myricetin twice a day for 16 weeks), STZ (fed a high-fat diet for 4 weeks, then treated with STZ for 5 days followed by intragastric administration of myricetin vehicle twice a day for 16 weeks), and myricetin + streptozotocin (MSTZ) (fed a high-fat diet for 4 weeks, then treated with STZ for 5 days, followed by intragastric administration of myricetin twice a day for 16 weeks). In detail, the DCM model was constructed by feeding mice with a high-fat diet (60% kcal fat) for 4 weeks, followed by overnight fasting and intraperitoneal injection with a dose of STZ (50 mg/kg × 5 days) dissolved in sodium citrate buffer (0.03 g/ml sodium citrate and 0.02 g/ml citric acid, pH = 4.2–4.5). After that, mice with blood glucose > 16.7 mmol/L were considered to have developed DM [25], and continuous high-fat diet feeding for 16 weeks was used to induce DCM.

**Fig. 1:**
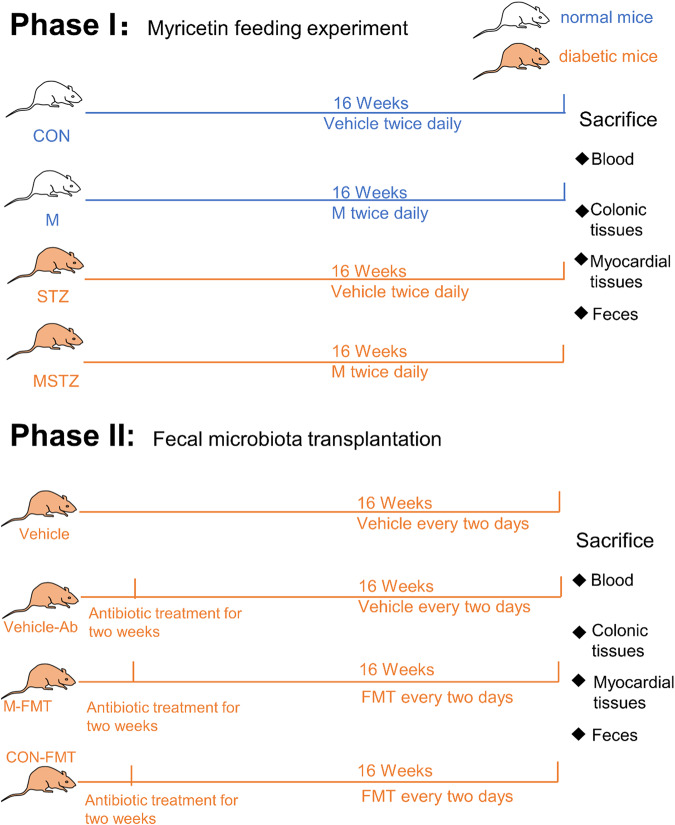
Flow diagram of the study design. The study was divided into two phases. In the first phase, 40 mice were randomly assigned into four groups: control group (CON), myricetin treatment group (M), streptozotocin treatment group (STZ), and myricetin and streptozotocin treatment group (MSTZ). Mice were fed a high-fat diet combined with low-dose STZ intraperitoneal injection to establish a diabetes model, and continuous high-fat diet feeding for 16 weeks was used to induce DCM. After 16 weeks of treatment with myricetin or myricetin vehicle, the mice were sacrificed and the blood, colonic tissues, myocardial tissues, and feces were harvested. In the second phase, another 40 DCM mice were divided into four groups: 10% glycerol solvent control group (Vehicle), 10% glycerol solvent antibiotic treatment control group (Vehicle-Ab), fecal microbiota transplantation group (gut contents derived from MSTZ mice, M-FMT) and fecal microbiota transplantation group (gut contents derived from CON mice, CON-FMT). After 16 weeks of treatment with fecal microbiota transplantation, the mice were sacrificed and the blood, colonic tissues, myocardial tissues, and feces were harvested identically.

The mice’s body weight and random blood glucose were measured every four weeks. During the last week of intervention, the feces were collected daily and stored at −80 °C until FMT. After that, all mice were euthanized by carbon dioxide inhalation. Serum samples were centrifuged for 5 min at 5000 rpm (rotor diameter 20 cm) at 4 °C, and the supernatants were then collected and stored at −80 °C. Each mouse’s heart and bowel tissues were divided into two parts: one was stored at −80 °C until processing, and the other was subjected to histopathological examination (Fig. 1).

### Fecal microbiota transplantation

The gut contents collected from the MSTZ and CON mice were weighed, homogenized, and suspended to a final concentration of 200 mg/ml, and centrifuged at 800 rpm (rotor diameter 20 cm) for 3 min in a saline solution containing 10% glycerol, then the bacterial suspension was stored at −80 °C. Before FMT, the content of myricetin in the bacterial suspension, prepared by the gut contents in the MSTZ group, was measured by HPLC (*λ* = 352 nm) to ensure no residue of myricetin remained.

Forty DCM mice established as described above were divided into four groups (*n* = 10 per group): vehicle (treated with 10% glycerol for 16 weeks intragastrically), vehicle + Abx (treated with10% glycerol for 16 weeks and antibiotics for 2 weeks intragastrically), M-FMT (treated with antibiotics for 2 weeks and gut contents derived from the above MSTZ mice were intragastrically administered every 2 days for 16 weeks), CON-FMT group (treated with antibiotics for 2 weeks and gut contents derived from CON mice were intragastrically administered once every 2 days for 16 weeks). An antibiotic cocktail was composed of ampicillin (1 g/L), vancomycin (0.5 g/L), neomycin sulfate (1 g/L), and metronidazole (1 g/L). The samples of blood, heart and bowel tissues, and feces were collected as described above after sacrificing the mice (Fig. 1).

### Histological analysis of paraffin sections

The mice’s heart and colon (2–4 cm above the cecum) were collected and prepared by standard methods for histopathological analysis. According to standard protocols, sections were stained with hematoxylin–eosin (H&E) and periodic acid-Schiff (PAS). The H&E staining was used to calculate the cardiomyocyte cross-sectional area (CSA) and observe the morphology of the striated muscle. PAS was used to identify the number of goblet cells in colon tissue. Immunohistochemistry (IHC) was performed to determine the amount of type I collagen. In brief, antigen retrieval was performed in sodium citrate buffer (pH = 6) in a microwave oven, and 8% goat serum was used to block nonspecific binding. After incubation with primary antibody [collagen I (1:500), ab90395, Abcam] overnight at 4 °C, sections were washed with PBS for 5 min × 3 times and then incubated with anti-mouse or anti-rabbit secondary antibody. After that, staining was visualized with DAB. Negative controls were performed with PBS instead of primary antibody. Finally, counterstaining was performed with Papanicolaou hematoxylin. Brown areas were considered positive. Image-Pro 6.0 (Media Cybernetics, Bethesda, MD, USA) was used to analyze images and calculate results.

### Assessment of cardiac structure and function

Two-dimensional echocardiography was performed on anesthetized (inhaled isoflurane) mice. Images were then analyzed by an observer blinded to the mouse group. Left ventricular ejection fraction (LVEF), fractional shortening (FS), left ventricular internal dimension diastole (LVIDd), and left ventricular internal dimension systole (LVIDs) were measured according to the images of three independent cardiac cycles acquired from each mouse.

### Detection of serum LPS

Serum LPS was measured using assay kits purchased from the Nanjing Jiancheng Bioengineering Institute (Nanjing, China), according to the manufacturer’s instructions.

### 16S rDNA gene high-throughput sequencing

Fecal microbial DNA was extracted from mouse feces using a Mag Pure Soil DNA LQ Kit. The quality of extracted DNA was evaluated by agarose gel electrophoresis. DNA sequences were amplified by polymerase chain reaction (PCR) and sequenced with an Illumina MiSeq. Operational taxonomic units (OTUs) were clustered with a 97% similarity cut-off using UPARSE software, and chimeric sequences were identified and removed using UCHIME. To obtain the species classification information corresponding to each OTU, the RDP Classifier algorithm (V16 http://rdp.cme.msu.edu/) was used for comparative analysis of representative OTU sequences. The species information of the community was annotated at various levels: kingdom, phylum, class, order, family, genus, and species; the correlation analysis of sample composition and community structure differences among samples was carried out.

### Western blotting analysis

Total protein was extracted from mice’s heart and colon tissues using radio-immunoprecipitation assay (RIPA) lysis buffer, and protein concentrations of the extracts were assessed by BCA assay. Western blotting was performed using the following antibodies: TLR4 (1:800, Abcam, ab- 13556), MyD88 (1:1000, Abcam, ab-219413), NF-κB p65 (1:10,000, Abcam, ab-32536), NF-κB p65 (phospho-T254) (1;800, Abcam, ab-131100), occludin (1:1000, Abcam, ab-216327), GAPDH (1:1000, Abcam, ab-32536), and β-actin (1: 500, Abcam, ab-115777). Band intensities were quantified using Image Pro Plus 6.0.

### Statistical analysis

Prism, version 8.0 (GraphPad) was used for the statistical analysis. Data are presented as mean ± standard error of the mean (SEM). Comparisons of multiple groups were tested using a one-way analysis of variance (ANOVA), followed by Tukey’s post-hoc test. A *P*-value < 0.05 was considered statistically significant.

### Study approval

All animal experiments were approved by the Animal Ethics Committee of Shantou University Medical College (No. SUMC2021-463).

## Results

### Myricetin ameliorates DCM-associated cardiac dysfunction and fibrosis

**Fig. 2:**
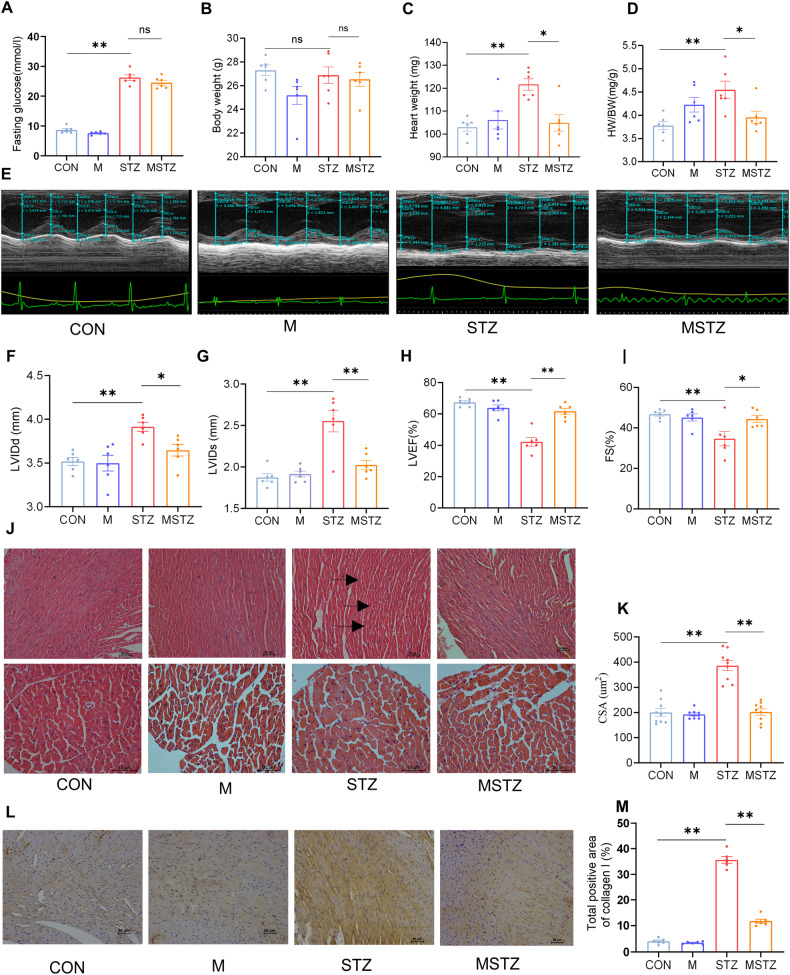
Evaluation of cardiac structure and function. **A** Blood glucose; **B** body weight (BW); **C** heart weight (HW); **D** HW/BW ratio; **E** M-mode echocardiography; **F** left ventricular internal dimension diastole (LVIDd); **G** left ventricular internal dimension systole (LVIDs); **H** left ventricular ejection fraction (LVEF); **I** fractional shortening (FS); **J** morphological changes of myocardial tissue shown by hematoxylin and eosin (H&E) staining. The black arrows indicate the disorganization of striated muscle. **K** The cross-sectional area (CSA) of cardiomyocytes; **L** immunohistochemistry (IHC) for collagen I; **M** quantitation of the collagen I. Data are presented as means ± SEM. One-way ANOVA was used to analyze statistical differences. NS for *P* > 0.05, *P < 0.05, **P < 0.01.

The blood glucose in STZ mice was higher than in CON mice (26.23 ± 0.93 mmol/L vs. 8.68 ± 0.47 mmol/L, *P* < 0.05), while no significant difference was found between STZ and MSTZ groups (Fig. 2A and Supplementary Table 1). There was no difference in body weight (BW) among the four groups (Fig. 2B and Supplementary Table 1). The heart weight (HW) (121.67 ± 2.46 vs. 103.00 ± 1.75, *P* < 0.05) and the HW/BW ratio (4.55 ± 0.19 vs. 3.78 ± 0.09, *P* < 0.05) in the STZ group were higher than that in the CON group, and decreased after treatment with myricetin (Fig. 2C, D and Supplementary Table 1). Compared with the CON or M group the parameters of cardiac function, including LVEF and FS, were reduced in the STZ group but were improved in the MSTZ group, which were opposite to LVIDs and LVIDd (*P* < 0.05) (Fig. 2F–I and Supplementary Table 2). The disordered myocardium, enlarged cardiomyocytes, and increased collagen I seen in the STZ group were not observed in the CON or M groups and were attenuated by myricetin treatment (*P* < 0.01; Fig. 2J–M).

### Myricetin alters the gut microbiota of DCM mice

**Fig. 3:**
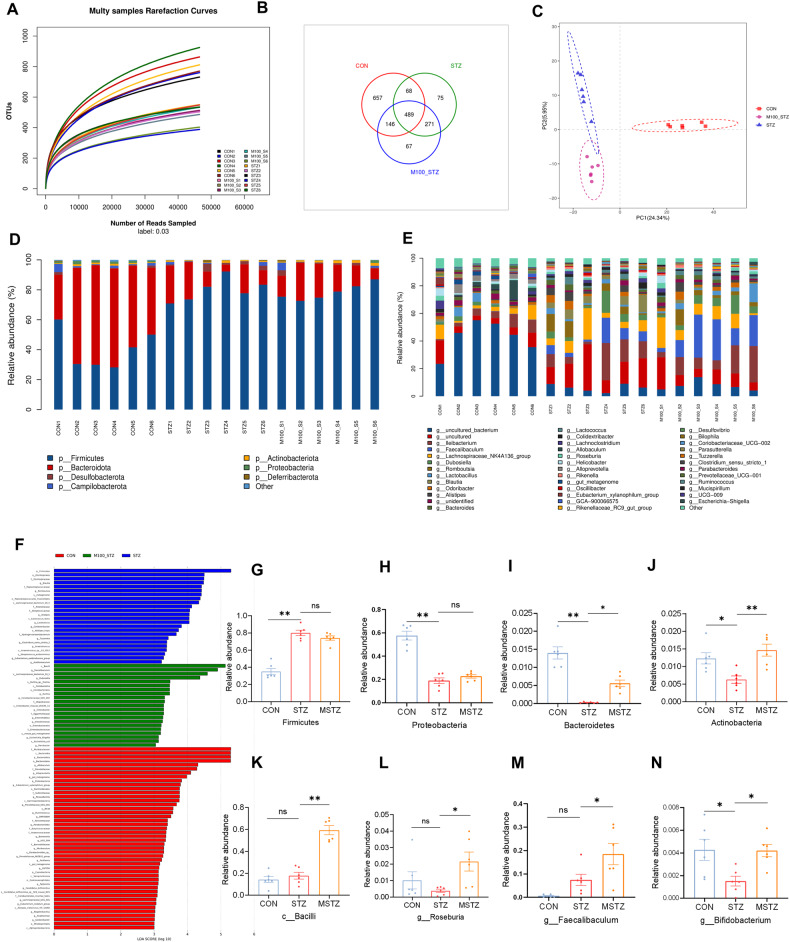
Composition of gut microbiota analyzed by 16S rRNA sequencing. **A** Rarefaction curves of bacterial OTUs; **B** difference of gut microbiota in the three groups shown by Venn diagrams; **C** partial least-squares-discriminant analysis (PLS-DA) analysis at the OTU level; **D** distribution of fecal microbiota at the phylum level; **E** distribution of fecal microbiota at the genus level; **F** biomarker taxa analyzed by LEfSe; **G** relative abundance of *Firmicutes*; **H** relative abundance of *Proteobacteria*; **I** relative abundance of *Bacteroidetes*; **J** relative abundance of *Actinomycetes*; **K** relative abundance of *Bacilli*; **L** relative abundance of *Roseburia*; **M** relative abundance of *Faecalibaculum*; **N** relative abundance of *Bifidobacterium*. Data are expressed as mean ± SEM. One-way ANOVA was used to analyze statistical differences. NS for *P* > 0.05, *P < 0.05, **P < 0.01.

The fecal samples were collected from CON, STZ, and MSTZ groups for 16S rDNA sequencing to clarify whether myricetin could alter gut microbiota composition. The number of operational taxonomic units (OTUs) reached saturation, as visualized by a rarefaction curve, suggesting the collected fecal samples were representative (Fig. 3A). The Venn diagram showed that a total of 1773 OTUs were obtained from the fecal samples in the three groups, of which 489 OTUs were shared by all three groups (Fig. 3B). Partial least squares-discriminant analysis (PLS-DA) indicated that the gut microbiota from the STZ group was distinctly different from the CON group but partially overlapped with the MSTZ group (Fig. 3C). Specifically, the relative abundance of predominant gut microbiota at the phylum (Fig. 3D, G–J) and genus (Fig. 3E, L–N) levels were compared among the three groups. At the phylum level, *Firmicutes* and *Bacteroidetes* were the leading phyla in all groups, occupying over 80% of the total sequences (Fig. 3D). The relative abundance of *Firmicutes* in the STZ group was higher than that in the CON group (*P* < 0.01; Fig. 3G), but the relative abundance of *Proteobacteria* in the STZ group was lower than that in the CON group (*P* < 0.01; Fig. 3H). *Firmicutes* and *Proteobacteria* were not statistically different between the STZ and MSTZ groups (*P* > 0.05; Fig. 3G, H). Compared with the CON group, *Bacteroidetes* and *Actinomycetes* were lower in the STZ group but increased in the MSTZ group (*P* < 0.05; Fig. 3I, J). Linear discriminant analysis effect size (LEfSe) indicated that the bacteria that changed the most were *Bacilli* (*P* < 0.05; Fig. 3F, K). At the genus level, the relative abundance of short-chain fatty acid (SCFA)-producing bacteria involving *Roseburia, Faecalibaculum,* and *Bifidobacterium* was higher in the MSTZ group when compared with the STZ group (*P* < 0.05; Fig. 3L–N).

### Myricetin improves the intestinal barrier of DCM

**Fig. 4:**
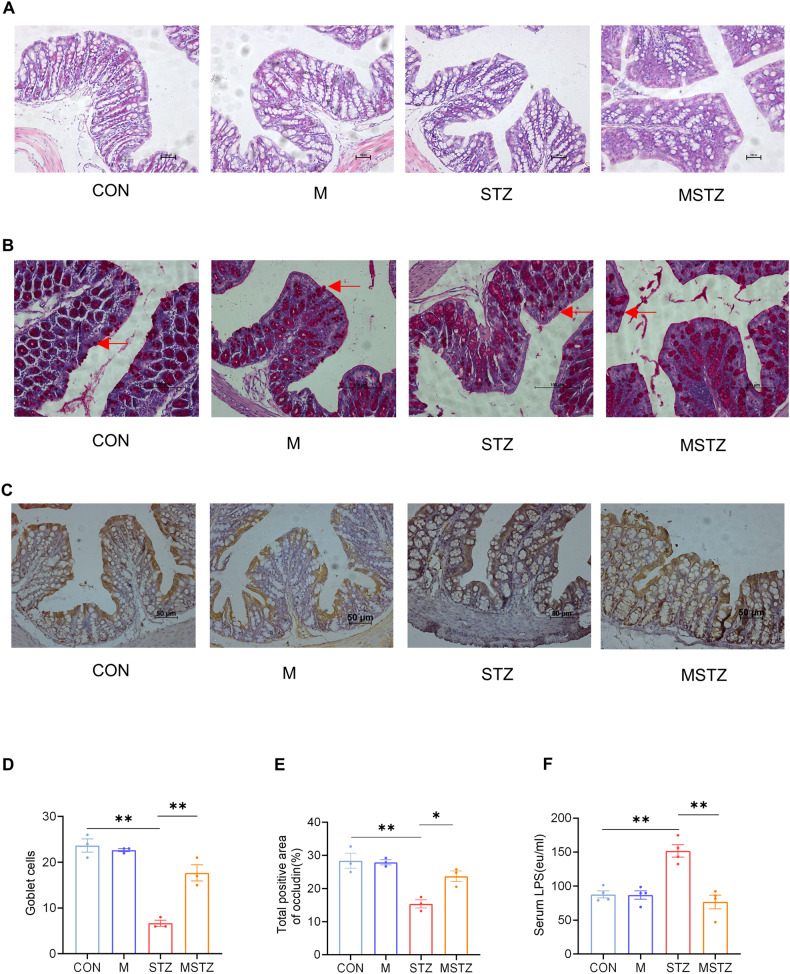
Assessment of intestinal barrier and endotoxemia. **A** Intestinal epithelial cells stained with H&E; **B** goblet cells stained (red arrows) with PAS, **C** occludin analyzed by IHC; **D** quantitation of occludin; **E** number of goblet cells per microscopic field (original magnification ×200) in the colon; **F** serum LPS detected by ELISA. Data are expressed as mean ± SEM. One-way ANOVA was used to analyze statistical differences. NS for *P* > 0.05, *P < 0.05, **P < 0.01.

H&E staining of colon tissue revealed a disordered arrangement and decreased density of intestinal epithelial cells in STZ mice, compared with CON or M mice, which were considerably improved in the MSTZ group (Fig. 4A). The number of goblet cells, measured by periodic acid-Schiff (PAS) staining (Fig. 4B, D), and the expression of occludin, measured by IHC (Fig. 4C, E), were decreased in the STZ group compared with the CON or M group, while elevated in the MSTZ group (*P* < 0.05). The serum level of LPS, detected by ELISA, was distinctly upregulated in the STZ group compared with the CON or M groups while downregulated in the MSTZ group (*P* < 0.01; Fig. 4F).

### Myricetin inhibits the TLR4/MyD88 pathway in DCM

**Fig. 5:**
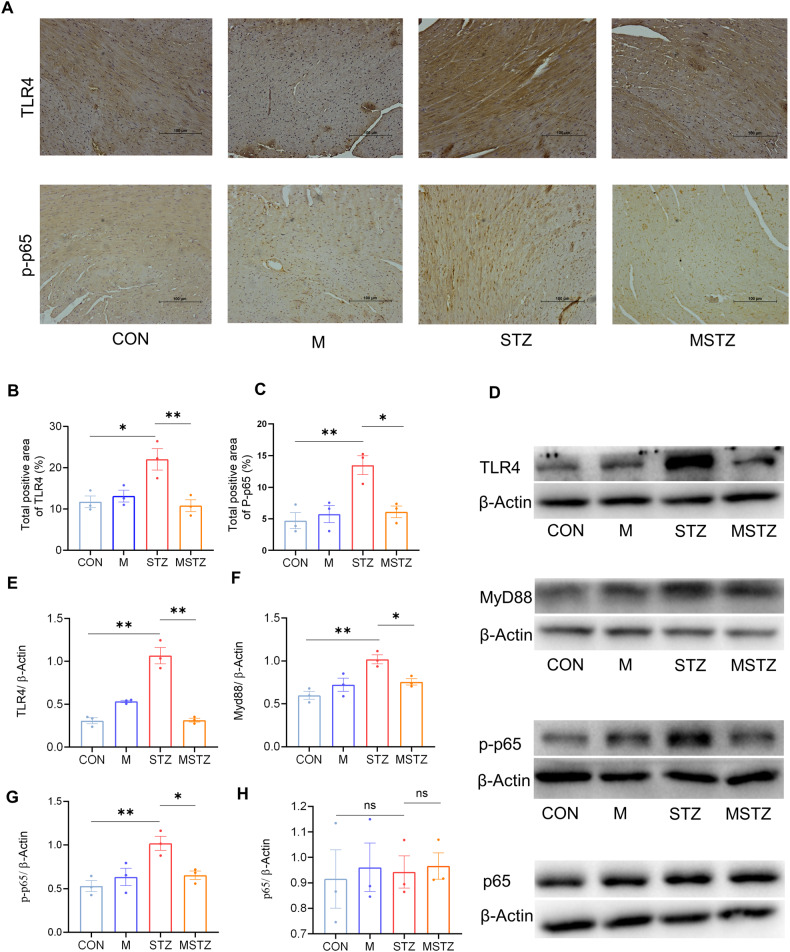
Myricetin alleviates the inflammatory response of cardiomyocytes in DCM mice. **A** Expression of TLR4 and p-p65 in myocardial tissues detected by IHC; **B** positive area of TLR4; **C** positive area of p-p65; **D** immunoblot analysis of TLR4/β-actin, MyD88/β-actin, p-p65/β-actin and total p65/β-actin in cardiomyocytes; **E** densitometric analysis of TLR4/β-actin in cardiomyocytes; **F** densitometric analysis of MyD88/β-actin in cardiomyocytes; **G** densitometric analysis of p-p65/β-actin in cardiomyocytes; **H** densitometric analysis of total p65/β-Actin in cardiomyocytes. Data are expressed as mean ± SEM. One-way ANOVA was used to analyze statistical differences. NS for *P* > 0.05, *P < 0.05, **P < 0.01.

To investigate the possible molecular mechanisms by which myricetin improves DCM through regulating gut microbiota, western blotting was applied to determine the expression of TLR4 and its downstream proteins involving MyD88, total p65, and phosphorylated p65 (p-p65) in myocardial tissues. TLR4, MyD88, and p-p65 expressions were increased in STZ mice compared with CON and M mice, but reversed by myricetin treatment (*P* < 0.05; Fig. 5D–G). Moreover, the IHC of TLR4 and p-p65 showed the same results (*P* < 0.05; Fig. 5A–C).

### FMT alleviates cardiac dysfunction and fibrosis in DCM

**Fig. 6:**
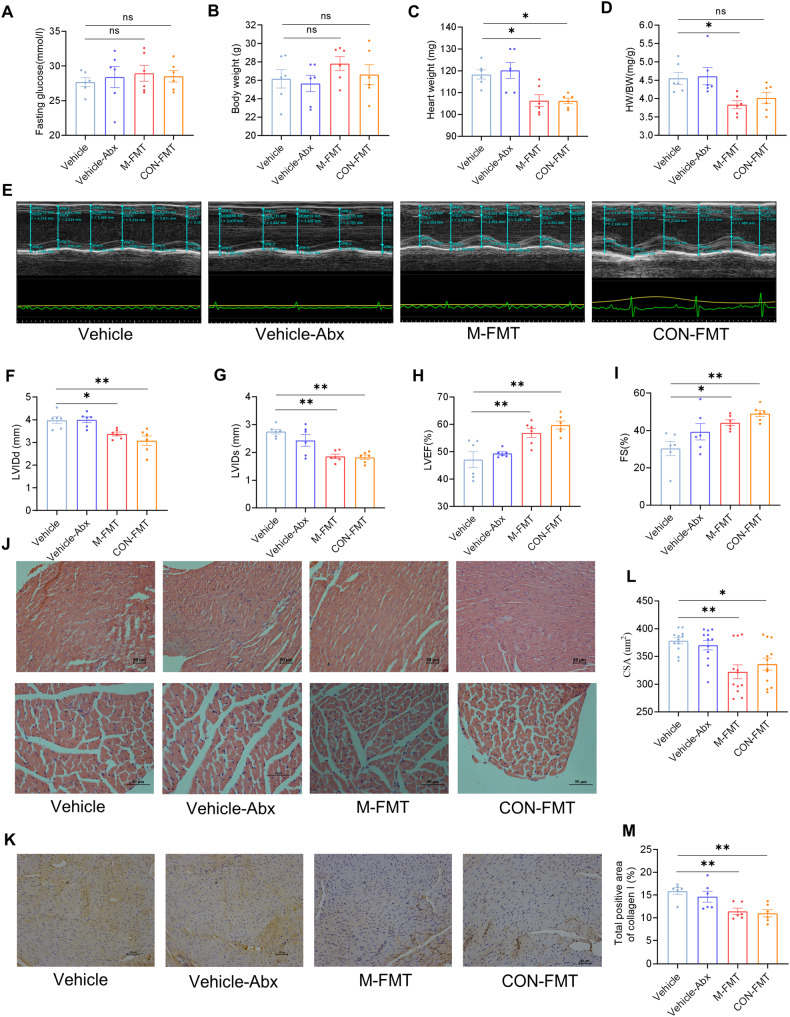
FMT attenuates cardiac remodeling and myocardial fibrosis in DCM mice. **A**–**D** Blood glucose, BW, HW, and HW/BW; **E** M-mode echocardiography; **F**–**I** LVIDd, LVIDs, LVEF, FS; **J** morphological changes of myocardial tissues stained with H&E; **K** cross-sectional area of cardiomyocytes (CSA); **L** IHC for collagen I; **M** collagen I-positive area. Data are presented as means ± SEM. One-way ANOVA was used to analyze statistical differences. NS for *P* > 0.05, *P < 0.05, **P < 0.01.

Neither the M-FMT nor CON-FMT groups had differences in blood glucose and BW compared with the Vehicle or Vehicle-Abx group (Fig. 6A, B and Supplementary Table 3). The HW (106.33 ± 2.70 mg vs. 118.33 ± 2.12 mg, *P* < 0.05) and HW/BW (3.83 ± 0.11 vs. 4.55 ± 0.16, *P* < 0.05) in the M-FMT group were reduced compared with the Vehicle group. However, the HW and HW/BW in the CON-FMT group were not statistically different from the Vehicle and Vehicle-Abx groups (Fig. 6C, D and Supplementary Table 3). Compared with the Vehicle or Vehicle-Abx group, the cardiac function evaluated by echocardiographic measurements of LVIDd, LVIDs, LVEF, and FS was improved in the M-FMT and CON-FMT groups (Fig. 6E–I and Supplementary Table 4). H&E staining showed that the myocardium in the Vehicle and Vehicle-Abx groups were hypertrophied, swollen, and arranged disorderly, but this pathological change was relieved in M-FMT and CON-FMT groups (*P* < 0.05; Fig. 6J, L). Moreover, the expression of collagen I measured by IHC in M-FMT and CON-FMT groups was decreased compared with the Vehicle and Vehicle-Abx groups (*P* < 0.05; Fig. 6K, M).

### FMT protects intestinal barrier integrity in DCM

**Fig. 7:**
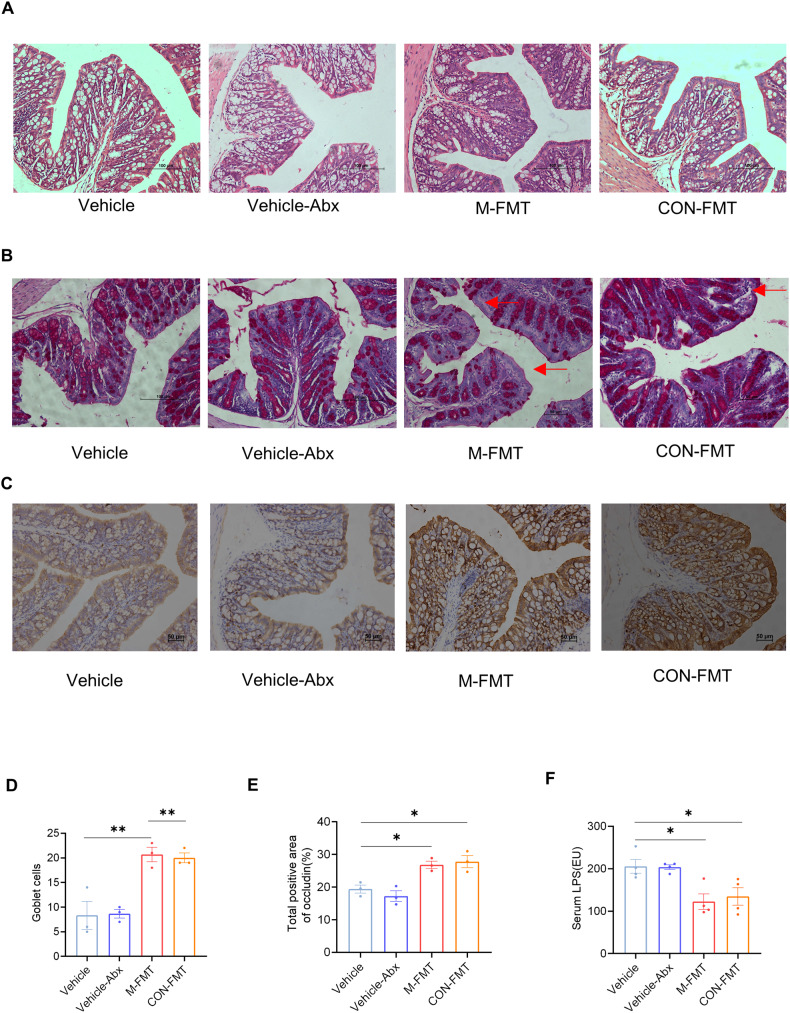
FMT attenuates damage to the intestinal barrier and metabolic endotoxemia in DCM mice. **A** Intestinal epithelial cells stained with H&E; **B** goblet cells (red arrows) stained with PAS; **C** occludin stained with IHC; **D** number of goblet cells per microscopic field (original magnification ×200) of the mouse colons; **E** occludin-positive area; **F** LPS measured by ELISA. Data are expressed as mean ± SEM. One-way ANOVA was used to analyze statistical differences. NS for *P* > 0.05, *P < 0.05, **P < 0.01.

The disordered arrangement and decreased density of intestinal epithelial cells in groups with FMT were alleviated compared with groups without FMT (Fig. 7A). Furthermore, the number of goblet cells (*P* < 0.05; Fig. 7B, D) and the expression of occludin (*P* < 0.05; Fig. 7C, E) in M-FMT and CON-FMT mice were increased compared with Vehicle and Vehicle-Abx mice. The serum LPS in the M-FMT and CON-FMT groups was decreased compared with the Vehicle and Vehicle-Abx groups (*P* < 0.05; Fig. 7F).

### FMT inhibits the TLR4/MyD88 pathway in DCM

**Fig. 8:**
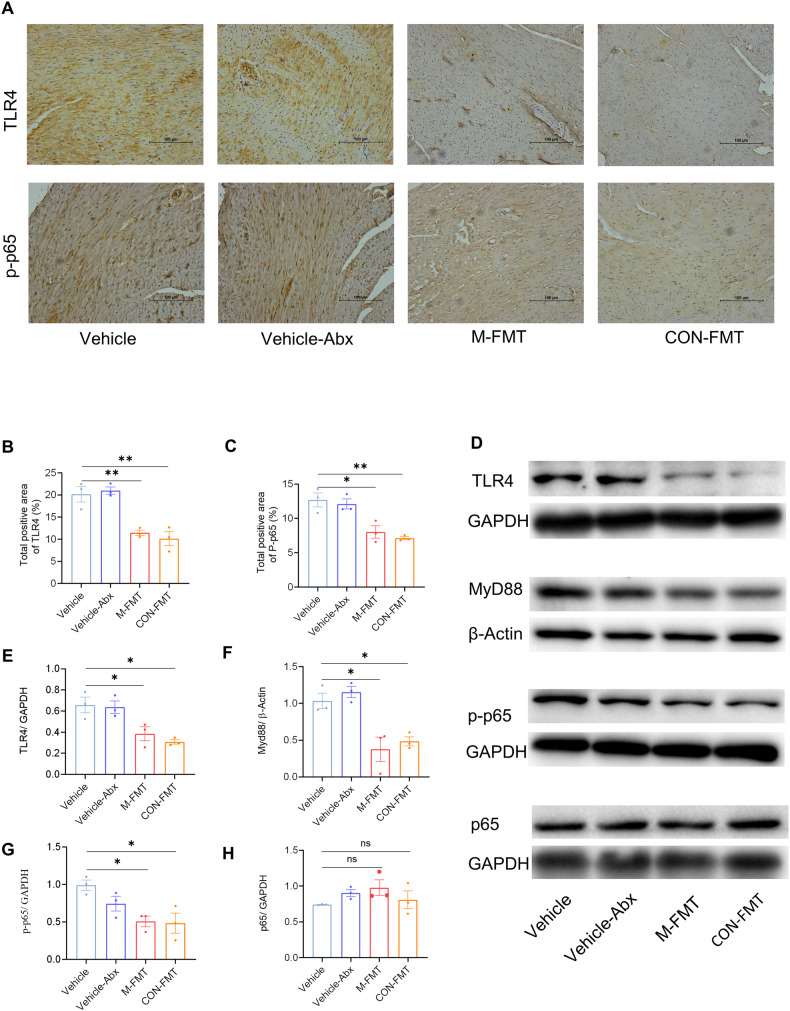
FMT alleviates the inflammatory response of cardiomyocytes in DCM mice. **A** Expressions of TLR4 and p-p65 in myocardial tissues detected by IHC; **B** positive area of TLR4; **C** positive area of p-p65; **D** immunoblot analysis of TLR4/GAPDH, MyD88/β-actin, p-p65/GAPDH and total p65/GAPDH in the cardiomyocytes; **E** densitometric analysis of TLR4/GAPDH in cardiomyocytes; **F** densitometric analysis of MyD88/β-actin in cardiomyocytes; **G** densitometric analysis of p-p65/GAPDH in cardiomyocytes; **H** densitometry analysis of total p65/GAPDH in cardiomyocytes. Data are expressed as mean ± SEM. One-way ANOVA was used to analyze statistical differences. NS for *P* > 0.05, *P < 0.05, **P < 0.01.

Lastly, we examined the expression of TLR4/MyD88 pathway-related proteins in myocardial tissue by western blotting and IHC. The expressions of TLR4 and its downstream proteins involving the MyD88 and the p-p65 in M-FMT or CON-FMT mice were detected by western blotting and found to be reduced compared with Vehicle or Vehicle-Abx mice (*P* < 0.05; Fig. 8D–H). Furthermore, the expressions of TLR4 and p-p65, as examined by IHC, were also consistent with western blotting (*P* < 0.05; Fig. 8A–C).

## Discussion

As a common complication of diabetes, DCM can impair the quality of life and increase mortality in patients with diabetes, as well as raise the risk of heart failure in patients without cardiovascular disease [4]. However, due to its complex pathological mechanism, DCM has no effective prevention and treatment strategies. Numerous studies have demonstrated that the gut microbiota in patients with diabetes has changed [26]. Myricetin, a natural polyphenolic compound in plants, shows therapeutic potential for DCM [20, 21] and can also regulate gut microbiota [27]. Nevertheless, the underlying mechanisms of myricetin have not been completely elucidated because of its low bioavailability. Therefore, we explored the possible role and mechanism of myricetin in DCM.

The study showed that myricetin alleviates cardiac dysfunction, myocardial fibrosis, and intestinal barrier disruption by regulating gut microbiota and its metabolites. Myricetin repaired the damaged intestinal barrier in DCM mice by increasing the abundance of SCFA-producing bacteria, lowering gut permeability and metabolic endotoxemia. The reduction of LPS in systemic circulation can inhibit TLR4 in cardiac tissue, reduce the translocation of NF-κB to the nucleus, and release inflammatory cytokines, thereby improving cardiac dysfunction and myocardial fibrosis in DCM. Furthermore, FMT experiments indicate that the gut contents derived from MSTZ and control mice prevent the progression of DCM. Thus, the study suggests that regulating gut microbiota is the main mechanism of myricetin in treating DCM.

The gut microbiota has shown severe dysbiosis in animals and patients with T2DM [28]. Our study confirms that the abundance of gut microbiota in DCM mice is distinctly decreased compared with control mice, and myricetin can significantly restore it. At the phylum level, *Firmicutes, Bacteroidetes, Proteobacteria*, and *Actinobacteria* are the predominant gut microbiota [29, 30], and they could reflect the changes in gut microbiota composition. In our study, the relative abundance of *Firmicutes* in DCM mice increased compared with control mice and is reversed by myricetin treatment. In addition, the abundance of *Bacteroidetes, Proteobacteria*, and *Actinobacteria* in DCM mice was reduced compared with the control mice and restored by myricetin treatment. We further analyzed the relative abundance of gut microbiota at the genus level. It was found that the abundance of probiotics is decreased in DCM mice, and recovered by myricetin, especially for SCFA-producing bacteria such as *Roseburia, Bifidobacterium*, and *Faecalibaculum* [28, 31, 32], which can use non-digestible carbohydrates to produce SCFAs. As a key regulator to enhance intestinal barrier function and communicate with the host immune system [33–36], SCFAs can prevent LPS from entering the systemic circulation, reducing metabolic endotoxemia.

Type 2 diabetes is characterized by chronic low-grade inflammation [37]. LPS, an essential component of Gram-negative bacteria cell walls, has been considered a major contributor to chronic low-grade inflammation [38]. Many studies have shown that the structure and function of the intestinal barrier in DM patients and animal models are impaired [39, 40]. LPS can enter the systemic circulation and activate a systemic inflammatory response when the intestinal barrier is impaired. Subsequently, it will be recognized by innate toll-like receptors (TLRs), especially TLR4, resulting in the translocation of p-p65 to the nucleus and the release of pro-inflammatory cytokines [38, 41, 42]. Besides inflammatory cells, TLR4 is also expressed in cardiomyocytes [43]. The role of TLR4-mediated inflammatory signaling in the development of DCM has been reported [18, 44–46]. Our study shows that serum LPS is increased in DCM mice, which is consistent with previous studies [47], and it can return to normal levels after treatment with myricetin. Furthermore, the expression of TLR4 and its downstream proteins MyD88 and p-p65 are increased in DCM and decreased by myricetin treatment. Based on these results, reducing TLR4/MyD88 pathway signaling in cardiomyocytes by reducing gut-derived LPS levels is one of the key mechanisms by which myricetin affects DCM.

To further test our hypothesis that the gut microbiota is a potential mechanism for the anti-DCM effects of myricetin, an FMT experiment was performed by transplanting the gut contents of mice treated with or without myricetin. Before FMT, HPLC was performed to ensure no residual myricetin in the bacterial suspension was transplanted into the recipient DCM mice. The gut contents from control mice were also transplanted into DCM mice to verify whether restoring normal gut microbiota could treat DCM. Pseudo-germ-free mice were established by antibiotic treatment for two weeks. At the same time, Vehicle and Vehicle-Abx groups were established to test whether antibiotics affected DCM-related phenotypes. Results showed that cardiac function and fibrosis and intestinal barrier function both in M-FMT and CON-FMT mice were improved, while the serum LPS and TLR4/MyD88 pathway-related proteins were reduced. Based on these results, our FMT experiments showed that myricetin could prevent DCM by changing or restoring microbiota and improving intestinal barrier function.

Our study demonstrates that gut dysbiosis and metabolic endotoxemia might be involved in the pathogenesis or development of DCM. Importantly, we found that myricetin treatment could inhibit DCM by increasing the abundance of SCFA-producing bacteria and decreasing serum LPS levels. These findings show a novel biochemical mechanism of myricetin in anti-DCM, laying the foundation for future myricetin-derived drugs.

### Supplementary information

Supplemental material

## Data Availability

All data generated or analyzed during this study are included in this article.
