# Supplementary material for: Myricetin alleviates diabetic cardiomyopathy by regulating gut microbiota and their metabolites
Source: Nutr Diabetes. 2024 Mar 12;14:10. doi: 10.1038/s41387-024-00268-4 (PMC10933338; doi:10.1038/s41387-024-00268-4)
Supplement: Supplementary file 1 — Supplemental material [file 41387_2024_268_MOESM1_ESM.docx]

**Supplemental material**

Supplementary Table 1: Effects of myricetin on fasting glucose, heart weight (HW), and body weight (BW). Data were presented as means ± SEM; **P* < 0.05, compared with CON or M group, and **^#^***P* < 0.05, compared with STZ group.

|  |  | CON | M | STZ | MSTZ |
| --- | --- | --- | --- | --- | --- |
| *n*  Fasting glucose (mmol/l)  BW (g)  HW (mg)  HW/BW (mg/g) | | 6 | 6 | 6 | 6 |
|  |  | 8.68±0.465 | 7.60±0.198 | 26.23±0.933* | 24.55±0.782 |
|  |  | 27.30±0.437 | 25.18±0.760 | 26.88±0.700 | 26.53±0.587 |
|  |  | 103.00±1.751  3.78±0.090 | 106.17±3.936  4.23±0.157 | 121.67±2.459*  4.55±0.185* | 104.83±3.61**^#^**  3.96±0.133**^#^** |

Supplementary Table 2: Effects of myricetin on echocardiographic parameters. LVIDd: left ventricular internal dimension diastole; LVIDs: left ventricular internal dimension systole; FS: fractional shortening; EF: LV ejection fraction. Data were presented as means ± SEM; **P* < 0.05 compared with CON or M group; **^#^***P* < 0.05 compared with STZ group.

|  |  | CON | M | STZ | MSTZ |
| --- | --- | --- | --- | --- | --- |
| *n*  LVIDd (mm)  LVIDs (mm)  EF (%)  FS (%) | | 6 | 6 | 6 | 6 |
|  |  | 3.52 ± 0.045 | 3.50±0.090 | 3.91±0.051* | 3.65±0.067**^#^** |
|  |  | 1.87±0.045 | 1.92±0.037 | 2.55±0.131* | 2.03±0.057**^#^** |
|  |  | 67.40±1.080  46.75±0.946 | 63.84±1.870  45.10±1.750 | 42.20±2.753*  34.67±3.607* | 62.61±1.887**^#^**  45.68±1.621**^#^** |

Supplementary Table 3: Effects of FMT on fasting glucose, heart weight (HW), and body weight (BW). Data were presented as means ± SEM; **P* < 0.05 compared with Vehicle or Vehicle-Abx; **^#^***P* < 0.05 compared with Vehicle or Vehicle-Abx.

|  |  | Vehicle | Vehicle-Abx | M-FMT | CON-FMT |
| --- | --- | --- | --- | --- | --- |
| *n*  Fasting glucose (mmol/l)  BW (g)  HW (mg)  HW/BW (mg/g) | | 6 | 6 | 6 | 6 |
|  |  | 26.35±1.120 | 28.40±1.543 | 30.22±1.186 | 28.52±0.816 |
|  |  | 26.15±1.003 | 25.63±0.890 | 27.80±0.77 | 26.62±1.084 |
|  |  | 118.33±2.124  4.55±0.164 | 120.17±3.655  4.61±0.233 | 106.33±2.704*  3.834±0.110* | 106.17±1.276  4.02±0.156 |

Supplementary Table 4: Effects of FMT on echocardiographic parameters. LVIDd: left ventricular internal dimension diastole; LVIDs: left ventricular internal dimension systole; FS: fractional shortening; EF: LV ejection fraction. Data were presented as means ± SEM; **P* < 0.05 compared with Vehicle or Vehicle-Abx; **^#^***P* < 0.05 compared with Vehicle or Vehicle-Abx.

|  |  | Vehicle | Vehicle-Abx | M-FMT | CON-FMT |
| --- | --- | --- | --- | --- | --- |
| *n*  LVIDd (mm)  LVIDs (mm)  EF (%)  FS (%) | | 6 | 6 | 6 | 6 |
|  |  | 3.98±0.149 | 3.99±0.127 | 3.38±0.086* | 3.08±0.212**^#^** |
|  |  | 2.75±0.085 | 2.43±0.210 | 1.86±0.082* | 1.83±0.080**^#^** |
|  |  | 47.11±2.883  30.34±3.816 | 49.39±0.597  39.28.73±4.418 | 56.90±1.708*  44.14±1.575* | 59.78±1.441**^#^**  49.08±1.677**^#^** |
